# The Role of HIF-1α in Retinopathy of Prematurity: A Review of Current Literature

**DOI:** 10.3390/jcm13144034

**Published:** 2024-07-10

**Authors:** Monika Modrzejewska, Oliwia Zdanowska, Piotr Połubiński

**Affiliations:** 1 2nd Department of Ophthalmology, Pomeranian Medical University in Szczecin, Powstańców Wielkopolskich 72, 70-111 Szczecin, Poland; 2 K. Marcinkowski University Hospital in Zielona Góra, 65-046 Zielona Góra, Poland; 3 Scientific Association of Students, 2nd Department of Ophthalmology, Pomeranian Medical University in Szczecin, Powstańców Wielkopolskich 72, 70-111 Szczecin, Poland

**Keywords:** HIF-1α, ROP, hypoxia-inducible factor, retinopathy of prematurity, potential target for new therapies of ROP, neovascularization, preterm infant

## Abstract

Hypoxia-inducible factor (HIF) plays a crucial role in regulating oxygen sensing and adaptation at the cellular level, overseeing cellular oxygen homeostasis, erythrocyte production, angiogenesis, and mitochondrial metabolism. The hypoxia-sensitive HIF-1α subunit facilitates tissue adaptation to hypoxic conditions, including the stimulation of proangiogenic factors. Retinopathy of prematurity (ROP) is a proliferative vascular disease of the retina that poses a significant risk to prematurely born children. If untreated, ROP can lead to retinal detachment, severe visual impairment, and even blindness. The pathogenesis of ROP is not fully understood; however, reports suggest that premature birth leads to the exposure of immature ocular tissues to high levels of exogenous oxygen and hyperoxia, which increase the synthesis of reactive oxygen species and inhibit HIF expression. During the ischemic phase, HIF-1α expression is stimulated in the hypoxia-sensitive retina, causing an overproduction of proangiogenic factors and the development of pathological neovascularization. Given the significant role of HIF-1α in the development of ROP, considering it as a potential molecular target for therapeutic strategies appears justified. This review synthesizes information from the last six years (2018–2024) using databases such as PubMed, Google Scholar, and BASE, focusing on the role of HIF-1α in the pathogenesis of ROP and its potential as a target for new therapies.

## 1. Introduction

Retinopathy of prematurity (ROP) is a vasoproliferative disease of the retina that primarily affects children born prematurely and is a leading cause of visual impairment and blindness in children worldwide. Advances in neonatal care have significantly increased the survival rates of premature infants, subsequently raising the number of children at risk of developing ROP. A 2023 study utilizing nationwide databases from the US reported a notable increase in the incidence of ROP over recent years; the percentage of premature infants diagnosed with ROP rose from 4.4% in 2003 to 8.1% by 2019 [1]. In Poland, the prevalence of ROP was reported at 15.1% between 2016 and 2019, slightly rising to 15.6% from 2012 to 2021 [2]. Similar high levels were observed in other European countries: 38% in Italy (2017–2020), 28.3% in the Netherlands (2017), and 23.8% in Portugal (2012–2020) [2]. Several factors contribute to the pathogenesis of ROP, with preterm birth, a low birth weight, and exposure to variable levels of exogenous oxygen being the most significant [3]. Although the current literature lacks definitive guidelines for a safe range of oxygen saturation, clinical trial data suggest that managing to avoid fluctuations in oxygen saturation can play a protective role in preventing ROP in preterm infants. Maintaining saturation levels at 90–95% is reportedly safer than keeping them at 85–89% [4]. In recent years, experimental research has been exploring the role of additional factors in the development and severity of ROP, including infections (such as systemic inflammation during the prenatal period and neonatal sepsis) [5], and disorders of carbohydrate metabolism in newborns, such as hyperglycemia, insulin resistance, and low levels of insulin-like growth factor (IGF1) [6].

The retinal vascular system begins to form at 16 weeks of fetal life and continues to develop until 40 weeks of gestation [7]. Premature birth interrupts this process, leaving the retinal vasculature immature along with the nonvascular zones. This exposes immature ocular tissues to elevated levels of exogenous oxygen and hyperoxia, which trigger oxidative stress. The result is an increase in the synthesis of reactive oxygen species (ROS) and the inhibition of hypoxia-inducible factors (HIF), as well as vascular endothelial growth factor (VEGF), IGF-1, and erythropoietin (EPO). Consequently, these changes lead to the obliteration of the retinal blood vessel lumen and widespread hypoxia within this ocular structure [7,8,9]. The following stage is an ischemic phase also known as the fibrovascular proliferation phase, which typically occurs around weeks 32–34 of the postconceptional age. In response to hypoxia in the retina, the body compensates by overproducing growth factors, particularly VEGF, whose synthesis is stimulated by the increased expression of HIF-1α. However, this leads to abnormal neoangiogenesis, where the newly formed vessels fail to properly nourish the retina. Instead, they grow into the vitreous body, potentially causing fibrosis and ultimately leading to traction and retinal detachment [10,11]. If ROP remains untreated, it can result in severe ametropia, strabismus, impaired retinal function, and, in the most severe cases, complete retinal detachment and blindness [12]. The downregulation of HIF-1α induced by hyperoxia plays a critical role in the initial phase of ROP, while its stimulation by hypoxia is crucial for the second phase [7]. Factor HIF-1 appears to be a promising molecular target for future therapeutic options of ROP.

To date, there have been few publications exploring the correlation between HIF-1α and ROP. A PubMed search spanning from 2001 to 5 August 2024, using the keywords H‘IF 1 alpha ROP, yielded only 65 articles. Consequently, the authors of this article conducted a review of the current literature covering the most recent six years (2018–2024), utilizing references cited in search engines such as PubMed, Google Scholar, and BASE. After excluding articles not directly related to the topic or available only in non-English languages, a total of 49 publications were selected for inclusion in this literature review.

## 2. Discussion

### 2.1. Currently Used Therapeutic Methods for Treating Rop

Current treatments for ROP include laser therapy and anti-VEGF injections. The efficacy of three anti-VEGF drugs—ranibizumab, bevacizumab, and aflibercept—is well documented [3]. A 2022 meta-analysis comparing these methods in treating premature infants with ROP found that the success rate of a single treatment was highest with laser therapy at 89%, followed by bevacizumab injections at 87%, aflibercept at 81%, and ranibizumab at 74%. Notably, for zone I ROP (an area with a radius of twice the distance between the optic nerve disc and the fovea), the efficacy of anti-VEGF treatments (bevacizumab at 91% and ranibizumab at 78%) surpassed that of laser therapy, which stood at 66% [13]. However, anti-VEGF therapy carries risks, including reactivation requiring repeat injections, the need for frequent follow-ups, and increased risks of conjunctival and retinal hemorrhage, elevated intraocular pressure, retinal and choroidal ischemia, worsening retinal traction, cataracts, intraocular inflammation, retinal detachment, and potential systemic effects. Rare but serious adverse events such as pulmonary maturation disorders, respiratory failure, thromboembolic complications, and liver and kidney dysfunction have been reported [14]. Conversely, laser therapy, while effective, involves long treatment durations, the need for anesthesia, and risks of an irreversible visual field loss, the development of high myopia, and retinal damage [3,15]. Given these risks, there is a pressing need for research into alternative treatments for ROP.

### 2.2. Hypoxia-Inducible Factor—HIF

HIF was first described in 1992 by researchers Semenza and Wang. During their molecular study of the human EPO gene and its response to hypoxia, they detected an unknown protein complex involved in EPO induction, later named hypoxia-inducible factor [16]. The HIF transcription factor is a heterodimeric complex that acts as a central regulator of oxygen sensing and adaptation at the cellular level. It controls cellular oxygen homeostasis, erythrocyte production, angiogenesis, and mitochondrial metabolism. This protein is composed of two subunits: α (found in three isoforms: HIF-1α, HIF-2α, and HIF-3α), which is oxygen-dependent and responds to hypoxic conditions by undergoing stabilization and transcriptional activation through interaction with coactivators, and β, which is detected in the nucleus independently of oxygen conditions [17]. In the intrauterine environment, characterized by hypoxic conditions, HIF-1α is stimulated and subsequently activates pro-angiogenic mechanisms. The main pro-angiogenic factor responsible for retinal angiogenesis is glycoprotein VEGF-A. This activation is crucial for physiological retinal development. As normal pregnancy progresses and hypoxic conditions diminish, the activation of HIF-1 and its target genes ceases [10]. 

#### 2.2.1. Factor HIF under Normoxic Conditions

**Figure 1:**
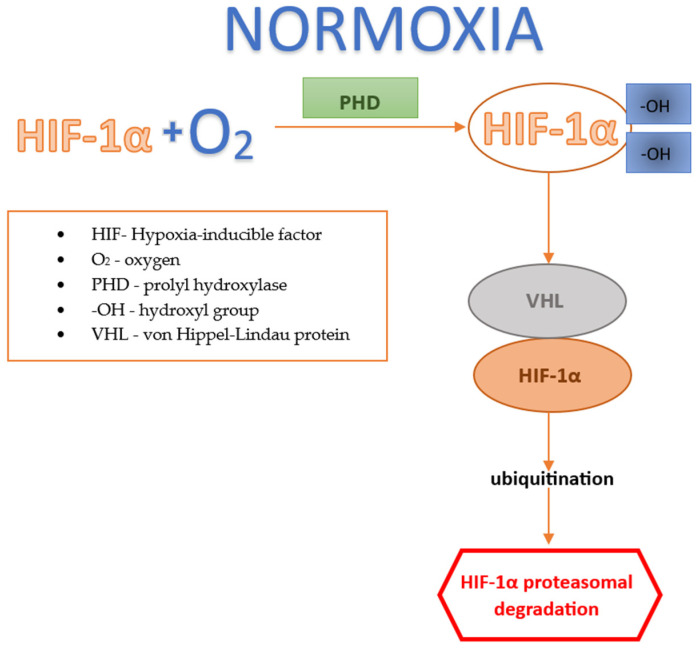
Factor HIF under normoxic conditions.

Under normoxic conditions, HIF-1α is hydroxylated in the cytosol by prolyl hydroxylase (PHD), leading to the α-ketoglutarate-dependent binding of HIF-1α to several components: the von Hippel–Lindau (VHL) protein, PHD, aspartate hydroxylase, and HIF inhibitory factor (FIH). This binding activates the ubiquitin ligase system, which targets HIF-1α for proteasomal degradation [18]; see Figure 1.

#### 2.2.2. Factor HIF under Hypoxic Conditions

**Figure 2:**
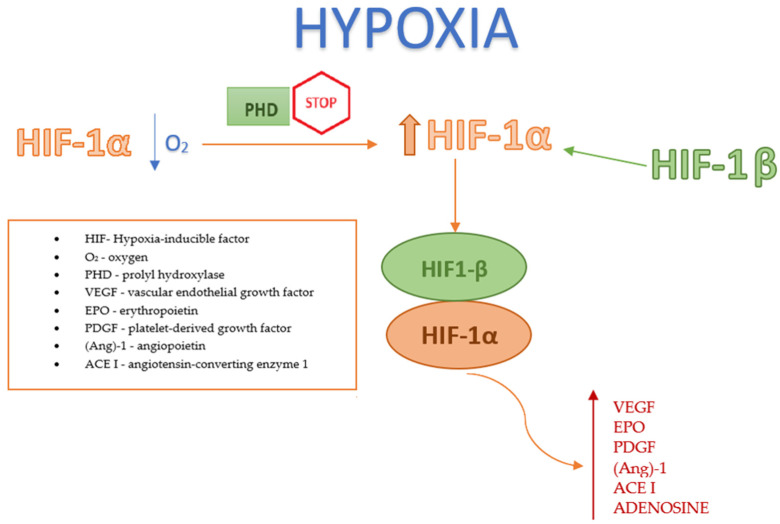
Factor HIF under hypoxic conditions.

Under hypoxic conditions, the enzymatic activity of PHD is inhibited (as oxygen and iron are essential cofactors for PHD), resulting in an increase in HIF-1α levels. This increase allows HIF-1α to combine with HIF-1β, aiding the body’s adaptation to hypoxia by initiating angiogenesis [19]; see Figure 2. Another pathway for increasing HIF-1α expression involves the inhibition of PHD due to impaired oxidative phosphorylation under hypoxia; this results from the inhibition of succinate dehydrogenase, leading to an accumulation of succinate that binds to the GPR91 receptor in the cytosol. Activation of this receptor suppresses PHD [20]. The HIF-1 complex facilitates tissue adaptation to hypoxic conditions by reprogramming the cellular metabolism (including glucose and iron handling), enhancing glycolysis, increasing mitochondrial NADPH synthesis, and stimulating the production of proangiogenic factors such as VEGF, EPO, platelet-derived growth factor (PDGF), angiopoietin (Ang)-1, angiotensin-converting enzyme 1, and adenosine [7].

### 2.3. HIF in Therapeutic Management

Several clinical studies have explored the stabilization of HIF under high oxygen concentrations as a means to inhibit neoangiogenesis and prevent the progression to the second phase of ROP. The therapeutic mechanisms outlined below present promising options for the future treatment of ROP in premature infants. 

#### 2.3.1. Role of Systemic Metabolism in the Protective Function of the Retina

##### Serine and 1-Carbon Metabolism

In 2019, Singh et al. [21] conducted an experimental study on newborn mice to analyze the metabolic processes triggered by HIF-1 activation in response to hypoxia and its protective role against oxygen-induced retinopathy (OIR), which is analogous to ROP. The study concluded that HIF-1 shifts the cellular/oxidative metabolism to glycolysis under hypoxic retinal conditions and regulates the urea cycle and serine/single-carbon metabolites supplied by the liver. The systemic stabilization of HIF, which depends on monocarbon metabolites, was identified as having a protective function against OIR. This dependency underscores the liver’s critical role in remote retinal protection. This discovery confirms the existence of a hepatic–eye axis, which allows protective action on retinal capillaries by stabilizing HIF in the liver, for example by suppressing HIF PHD in the liver, which has already been described in other publications [22]. The study also found that serine supplementation alone is insufficient for protective effects against OIR development, as it is metabolized too quickly; effective protection also requires the activation of HIF-1-dependent enzymes [21,23].

##### Fibrates

Fibrates, as peroxisome proliferator-activated receptor type α (PPAR-α) agonists, were studied for their effects on retinal conditions in 2019 by Tomita et al. [24]. They demonstrated the protective effects of Pemafibrate against pathological retinal neovascularization in mouse models of OIR. Pemafibrate, by increasing PPARα gene expression, led to elevated plasma levels of FGF21 secreted by hepatocytes and reduced plasma levels of HIF-1α along with VEGF-A [25]. Notably, Pemafibrate primarily affects the liver, where FGF21 production suppresses retinal HIF-1α expression. The study also compared the effects of Pemafibrate with those of Fenofibrate, another PPARα and PPARδ agonist. Pemafibrate was found to be more specific and effective in preventing retinal neovascularization. Unlike Fenofibrate, which is excreted through the kidneys, Pemafibrate is metabolized in the liver, making it suitable for patients with renal impairment [24,26].

#### 2.3.2. HIF Inhibitors

##### Thrombomodulin 1

In 2021, Huang et al. [27] investigated the effects of recombinant thrombomodulin domain 1 (rTMD1) on pathological neovascularization in an animal model of OIR and its effects on conditions that faithfully mirror ROP. The study demonstrated that rTMD1 inhibits pathological neoangiogenesis and inflammation in OIR while preserving physiological vascular growth. This process occurs through the inhibition of HIF-1α, resulting in a consequent reduction in VEGF levels and the inhibition of angiogenesis. Intercellular adhesion molecule-1 and interleukin-6 levels were also decreased, producing an anti-inflammatory effect. These findings suggest that rTMD1 may be a potential therapeutic agent for the treatment of pathological neovascularization in ROP, among other conditions [27].

##### Topotecan, Doxorubicin

In 2019, Miwa et al. [28] conducted a study using a mouse model of OIR and suggested that HIF inhibition might be a viable therapeutic approach to prevent pathological angiogenesis and retinal neurodegeneration while preserving physiological VEGF levels. The study evaluated two HIF inhibitors with distinct mechanisms of action: topotecan, a topoisomerase inhibitor chemotherapeutic that inhibits HIF-1α protein accumulation, and doxorubicin, a cytostatic anthracycline antibiotic that prevents HIF-1α activation in response to hypoxia. Both agents were found to significantly inhibit pathological neovascularization in OIR. Additionally, topotecan was noted to reduce the visual impairments observed in OIR [28].

##### Decapterus Tabl Substrate

Due to the fact that most HIF inhibitors are cytostatics, which can also harm healthy, rapidly dividing cells, their use carries a high risk of numerous side effects. In 2020, Shoda et al. [29] identified six marine fish species that exhibit inhibitory effects on the HIF factor. Additionally, it was noted that components from Decapterus tabl in a mouse model of OIR suppressed retinal neovascularization by inhibiting HIF expression. Further research is required to develop similar therapeutic agents. The data suggest that such agents could represent a promising direction for treating ROP with a much better safety profile compared to existing chemotherapeutics [29].

CBP/p300-Interacting Transactivator with Glu/Asp-Rich C-Terminal Domain 2 (CITED2)-peptide Derived from the Intrinsically Disordered Protein CITED2

In their 2020 study, Usui-Ouchi et al. [30] demonstrated that the CITED2 peptide fragment regulates pathological angiogenesis in mouse models of OIR. Following intravitreal injection, the CITED2 peptide significantly reduced vascular obliteration and retinal neovascularization. The CITED2 protein, a cAMP-responsive transcriptional modulator, competes with HIF for binding to CBP/p300 under hypoxic conditions, thereby inhibiting HIF’s transcriptional activity, which relies on its interaction with these transcriptional coactivators. In the study, retinal cells absorbed the injected CITED2 peptide, leading to the local suppression of HIF gene expression and the prevention of retinal neovascularization. When comparing the efficacy of CITED2 to anti-VEGF therapy, it was found that, unlike Aflibercept, CITED2 not only prevented vascular obliteration but also suppressed pathological neovascularization during OIR [30].

##### Celastrol (Tripterine)

In their 2022 study, Zhao et al. [31] demonstrated for the first time that celastrol, by inhibiting the miR-17/HIF-1α/VEGF signaling axis, protects the glial cell function and reduces neovascularization and inflammation in the retina in a mouse model of OIR. Celastrol, a compound extracted from the roots of Tripterygium wilfordii and Tripterygium regelii, belongs to the quinone methide family and exhibits antibacterial, antioxidant, anti-inflammatory, anticancer, and insecticidal properties [32,33,34,35,36]. In this study, celastrol was shown to suppress HIF-1α expression, thereby inhibiting VEGF. This suppression contributes to the inhibition of the Akt/mTOR/p70S6K signaling pathway, leading to reduced neoangiogenesis and the enhanced protection of retinal microcirculation during OIR. Under hypoxic conditions, the retina typically accumulates microglia that attach to retinal capillaries to stimulate angiogenesis [37]. However, celastrol therapy also led to reduced levels of pro-inflammatory cytokines, including IL-1, IL-6, TNF-α, and MCP-1, as well as the enzymes COX2 and MMP9, preventing the activation of retinal microglia despite the hypoxia. These findings highlight the therapeutic potential of celastrol in ROP [31].

##### 3-Hydroxypyruvate

In 2018, Singh et al. [38] first described the action mechanism of 3-hydroxypyruvate as a hyperoxia-induced metabolite that inhibits neoangiogenesis by destabilizing HIF in a mouse model of OIR. The study showed that destabilization of HIF occurs through an increase in alpha-ketoglutarate (αKG) levels in response to 3-hydroxypyruvate. αKG serves as a cofactor for the hydroxylation of HIF-1α by PHD, enabling its binding to the VHL protein even under hypoxic conditions, ultimately leading to the proteasomal degradation of HIF. Additionally, it was confirmed both in vitro and in vivo that while HIF stabilization increases serine synthesis, the accumulation of 3-hydroxypyruvate and the resulting destabilization of HIF lead to a decrease in serine synthesis during hyperoxia [38].

#### 2.3.3. Apurinic/Apyrimidinic Endonuclease 1/Oxidation-Reduction Factor 1 (APE1/Ref-1)

APE1/Ref-1 protein has emerged as a potential therapeutic target for neovascular eye diseases, including ROP. APE1/Ref-1 inhibitors regulate the activation of transcription factors, thereby modulating angiogenesis, inflammation, and oxidative stress responses [39]. APE1/Ref-1 is expressed in various cell types within the eye, such as retinal and choroidal endothelial cells, retinal pigment epithelial cells, and pericytes, and its expression intensifies in tissues with active inflammation [40]. In 2021, Hartman et al. [41] explored the anti-angiogenic effects of APE1/Ref-1 inhibitors—APX3330, APX2009, and APX2014—in the retina and choroid in an in vitro study. In a mouse model, these inhibitors demonstrated dose-dependent and route-dependent inhibitory effects on the retinal and choroidal vascular endothelium. Even a single intravitreal injection of a small volume (20 µM) of APX3330 reduced neovascularization. However, using higher doses of 25–50 mg/kg administered twice daily via gastric probe for 2 weeks significantly reduced lesion severity (by more than half). The oral route of administration showed better effects than intravitreal injections, leading the study authors to suggest that APE1/Ref-1 redox inhibitors should be considered potential oral therapeutic agents for the treatment of neovascular eye disease. APE1/Ref-1 has a redox function, meaning that through thiol–disulfide exchange it induces certain transcription factors, including HIF-1, which then generates the expression of genes such as VEGF and carbonic anhydrase 9, stimulating retinal neovascularization. APE1/Ref-1 inhibitors block redox activation, thus performing anti-angiogenic and anti-inflammatory functions [41].

#### 2.3.4. MicroRNA

MicroRNAs are non-coding small RNAs, 18–22 nucleotides in length, that bind to target regions of mRNAs to either repress translation or promote degradation. These microRNAs are expressed in most mammalian tissues and play crucial roles in fundamental biological processes, including brain and retinal neurodevelopment [42]. In a 2020 study, Guan et al. [43] identified miR-18a-5p as a new therapeutic target for pathological ocular neovascularization, which complicates conditions including ROP. It has been shown that miR-18a-5p is strongly expressed in embryonic retinas and that its levels decrease during development. However, in mice with OIR, the levels of miR-18a-5p were higher compared to those without the pathology. Intravitreal administration of an miRNA mimic, agomiR-18a-5p, led to the inhibition of pathological neoangiogenesis. This effect was mediated by the downregulation of fibroblast growth factor 1 (FGF1) and HIF-1α, and the suppression of the functions of retinal microvascular endothelial cells, including their migration and proliferation [43]. 

#### 2.3.5. Lowering Intraocular Pressure Reduces HIF-1α

In 2023, Yang et al. [44] published a research paper suggesting that lowering the intraocular pressure (IOP) may have a therapeutic effect on the development of retinal neovascular disease. The study assessed the effects of vitreous body injections using needles of different sizes (0.21 mm, 0.3 mm, 0.5 mm) in a mouse model of OIR. It was found that a puncture with a 0.5 mm diameter needle, as opposed to smaller needles, caused a sudden drop in IOP due to the leakage of vitreous fluid. This drop led to improved perfusion and an increase in retinal oxygen partial pressure, which in turn promoted the suppression of HIF-1α and proangiogenic factors like VEGF, inhibiting pathological neovascularization. Additionally, the study examined the effects of IOP-lowering drugs, Azarga and Travatan, administered as conjunctival sac drops on OIR. Both drugs inhibited angiogenesis by approximately 60% and reduced the sterile area. These findings suggest a new therapeutic approach to neovascular diseases based on managing IOP [44].

#### 2.3.6. Bone Marrow Cells

In their 2018 study, Babaei et al. [45] analyzed the levels of bone marrow-derived endothelial progenitor cells (EPCs) in premature infants with ROP and investigated their role in the disease’s pathogenesis. The study found a statistically higher number of EPCs in infants with ROP compared to a control group, which included healthy preterm and term-born infants. Furthermore, samples containing EPCs collected from the affected infants showed increased levels of HIF-α and VEGF upon molecular analysis. The elevated levels of EPCs and pro-angiogenic factors in children with ROP suggest that EPCs contribute significantly to the disease’s development [45].

Villacampa et al. [46] explored the impact of myeloid cells on HIF stabilization in a mouse model of OIR and its subsequent effects on retinal capillaries. They found that under post-birth physiological conditions, stimulation of the HIF pathway in myeloid cells is crucial for the development of a normal vascular bed. Myeloid cells aid in endothelial cell fusion during vessel formation and produce proangiogenic factors. In their study, an OIR model was utilized where mice had a marrow cell-specific deletion of Vhl, HIF-1α, and/or Epas1 (which encodes HIF-2α). The authors demonstrated that stabilizing both HIF-1α and HIF-2α in myeloid cells through VHL deletion significantly enhances the local expression of VEGF and bFGF, contributing to retinal vascular regeneration during ischemic retinopathy [46].

### 2.4. HIF-1α in the Diagnostic Management of Rop

Uddin et al. [47] described a potential technique for tracking and predicting the onset and progression of hypoxia-induced retinal neovascularization. In their study, the authors utilized a novel nanoparticle to selectively visualize HIF-1α mRNA in microglia/macrophages in a mouse model of OIR. This method involved a probe made of antisense short-hairpin RNA conjugated to diacyl-lipids (lipid AS-shRNA). Although the role of macrophages in retinal neovascularization is not fully understood, it is known that the induction of HIF-1α in monocytes that differentiate into macrophages promotes neoangiogenesis [48,49]. The findings of Uddin et al. suggest that lipid AS-shRNA can selectively bind to monocytes and be transported to the OIR retina, where it integrates into neovascular lesions, making them visible. This method of monitoring neovascularization through the molecular tracking of specific cells could revolutionize the diagnosis and treatment of proliferative retinopathy, including complications associated with ROP, among other diseases [47].

We summarize the results of the cited studies in Table 1 to better clarify the literature discussed above.

## 3. Conclusions

ROP is a leading cause of visual impairment and blindness in children worldwide. Despite significant advances in treatments for severe ROP, such as laser therapy and anti-VEGF injections, these therapies carry risks of side effects and disease recurrence, making ROP an ongoing focus of research. HIF-1α is recognized as a crucial factor in both the first and second phases of ROP pathogenesis, as reported in the literature. Therefore, its stabilization is seen as a promising target for future therapeutic strategies. Studies in the literature have explored various methods of HIF stabilization to inhibit neoangiogenesis and ROP progression. These methods include the inhibition of HIF, PHD, non-coding RNA function, APE1/Ref-1 proteins, and the influence of bone marrow cells and hepatic metabolism on HIF expression. Some studies also suggest a protective role of reduced IOP in the development of neovascular diseases. To date, most published experimental studies have utilized animal models of OIR, which are believed to closely replicate the conditions of ROP in premature infants. However, further extensive studies are necessary to fully decipher the complex molecular mechanisms underlying ROP’s pathogenesis and to evaluate the potential of HIF as a molecular therapeutic target in infants with ROP.

The above literature review summarizes the current hypotheses and experimental studies on utilizing the biochemical functions of the HIF factor in planning therapeutic options for neovascular diseases, particularly in the context of ROP. At present, there are very few articles discussing the importance of the HIF-1 factor in ocular neovascular diseases, in particular regarding the use of HIF-1 as a potential molecular target for ROP.

## Figures and Tables

**Table 1:** Potential role of HIF-1a in ROP management—summary of preclinical studies.

Authors	Year of Publication	Pharmacological Agent/Molecular Pathway	Animal Model Used during Research	Key Findings for Each Potential Therapeutic Agent
Singh et al. [21]	2019		Newborn mice models of OIR	HIF-1 regulates the transition of cellular metabolism to glycolysis under conditions of retinal hypoxia HIF-1 mediates the regulation of the urea cycle and serine/single-carbon metabolites provided by the liver Key importance of the liver for distant retinal protection
Tomita et al. [23]	2019	Pemafibrate	Mouse models of OIR	Protective effect of Pemafibrate on pathological retinal neovascularization The liver is the target site of Pemafibrate’s uptake Pemafibrate exhibits a suppressive effect on HIF-1α expression in the retina
Huang et al. [27]	2021	Recombinant Thrombomodulin 1	Mouse models of OIR	Recombinant Thrombomodulin 1 inhibits pathological neoangiogenesis and inflammation in OIR This process occurs through the inhibition of HIF-1α
Miwa et al. [28]	2019	Topotecan, Doxorubicin	Mouse model of OIR	Suppression of HIF—a potential therapeutic mechanism inhibiting pathological angiogenesis and retinal neurodegeneration Topotecan, Doxorubicin—HIF inhibitors—both prevented pathological neovascularization in OIR
Shoda et al. [29]	2020	Components of Decapterus tablets	Mouse model of OIR	Six species of marine fish have been discovered to exhibit inhibitory effects on the HIF factor Components of Decapterus tablets inhibited retinal neovascularization in OIR
Usui-Ouchi et al. [30]	2020	CITED2 peptide	Mouse model of OIR	The CITED2 peptide inhibited HIF expression, thereby preventing retinal neovascularization in an OIR model
Zhao et al. [31]	2022	Celastrol	Mouse model of OIR	Celastrol has a suppressive effect on HIF-1α Celastrol inhibits the miR-17/HIF-1α/VEGF axis, thereby preventing neurodegeneration and neovascularization, and reducing inflammation in the retina of OIR
Singh et al. [38]	2018	3-hydroxypyruvate	Mouse model of OIR	3-hydroxypyruvate inhibits neoangiogenesis by destabilizing HIF in the retina of OIR
Hartman et al. [41]	2021	Apurinic/Apyrimidinic Endonuclease 1/Reduction-Oxidation Factor 1 (APE1/Ref-1)	Mouse model of OIR	APE1/Ref-1 functions as a redox transcription activator APE1/Ref-1 regulates, among other things, the transcriptional activity of the HIF-1α factor Activated HIF-1α induces the expression of genes that stimulate neovascularization Blocking the redox activity of APE1/Ref-1 can counteract angiogenesis, inflammation, and the response to oxidative stress
Guan et al. [43]	2020	miRNA mimic, agomiR-18a-5p		miR-18a-5p as a novel potential therapeutic target for pathological ocular neovascularization Intravitreal injection of an miRNA mimic, agomiR-18a-5p, resulted in a decrease in HIF-1α and fibroblast growth factor levels, as well as the inhibition of neovascularization in the retina of OIR
Yang et al. [44]	2023	Reduced intraocular pressure (IOP)	Mouse model of OIR	The potential therapeutic impact of reduced IOP on the development of retinal neovascular diseases Under conditions of reduced IOP, the activity of HIF-1α is inhibited, which blocks the expression of proangiogenic factors, thereby slowing down pathological neovascularization
Villacampa et al. [46]	2020	Stabilizing HIF through VHL deletion	Mouse model of OIR	The study demonstrated that stabilizing HIF-1α and HIF-2α in bone marrow cells through VHL deletion stimulates the local expression of VEGF and bFGF, contributing to retinal vessel regeneration during ischemic retinopathy

## Data Availability

No new data were created or analyzed in this review. Data sharing does not apply to this article.
